# Can flat-detector CT after successful endovascular treatment predict long-term outcome in patients with large vessel occlusion? An Alberta Stroke Programme Early CT Score–based study

**DOI:** 10.1007/s10072-022-06511-z

**Published:** 2022-11-26

**Authors:** Michael Knott, Philip Hoelter, Stefan Hock, Iris Mühlen, Stefan T. Gerner, Maximilian I. Sprügel, Hagen B. Huttner, Stefan Schwab, Tobias Engelhorn, Arnd Doerfler

**Affiliations:** 1 Department of Neuroradiology, Friedrich-Alexander-Universität Erlangen-Nürnberg (FAU), Schwabachanlage 6, 91054 Erlangen, Germany; 2 Department of Neurology, Friedrich-Alexander-Universität Erlangen-Nürnberg (FAU), Schwabachanlage 6, 91054 Erlangen, Germany

**Keywords:** Stroke, Flat-detector CT, ASPECTS, Endovascular treatment

## Abstract

**Purpose:**

Recent studies postulate a high prognostic value of the Alberta Stroke Programme Early CT Score (ASPECTS) applied on non-contrast whole-brain flat-detector CT (FDCT) after successful endovascular treatment (EVT). The aim of this study was the evaluation of long-term patient outcome after endovascular treatment using postinterventional FDCT.

**Methods:**

Using a local database (Stroke Research Consortium in Northern Bavaria, STAMINA), 517 patients with successful endovascular treatment (modified Thrombolysis in Cerebral Infarction (mTICI) ≥ 2B) due to acute ischaemic stroke (AIS) and large vessel occlusion (LVO) of the anterior circulation were recruited retrospectively. In all cases, non-contrast FDCT after EVT was analysed with special focus at ASPECTS. These results were correlated with the functional outcome in long-term (modified Rankin Scale (mRS) shift from pre-stroke to 90 days after discharge).

**Results:**

A significant difference in FDCT-ASPECTS compared to the subgroup of favourable vs. unfavourable outcome (Δ mRS) (median ASPECTS 10 (10–9) vs. median ASPECTS 9 (10–7); *p* = 0,001) could be demonstrated. Multivariable regression analysis revealed FDCT-ASPECTS (OR 0.234, 95% CI − 0.102–0.008, *p* = 0.022) along with the NHISS at admission (OR 0.169, 95% CI 0.003–0.018, *p* = 0.008) as independent factors for a favourable outcome. Cut-off point for a favourable outcome (Δ mRS) was identified at an ASPECTS ≥ 8 (sensitivity 90.6%, specificity 35%).

**Conclusion:**

For patients with LVO and successful EVT, FDCT-ASPECTS was found to be highly reliable in predicting long-term outcome.

## Introduction

Endovascular treatment (EVT) has been proven to be the treatment of choice for patients with acute ischemic stroke (AIS) caused by large vessel occlusion (LVO) [1–3].

To screen for haemorrhagic complications after EVT, it is well-established to conduct a non-contrast whole-brain flat-detector CT (FDCT) to monitor the patient directly after this treatment [4–6]. FDCT has shown its ability not only to detect haemorrhage but also to visualise infarct demarcation by applying only a moderate amount of radiation dose [5, 7–10].

On the other hand, the Alberta Stroke Programme Early CT Score (ASPECTS) is a well-established scoring system for quantitative assessment of infarct extension as well as an early tool for prediction of long-term outcome. Based mainly on non-enhanced conventional CT images, it is in clinical and radiological practise applied as a standard scoring system for the evaluation of patients with AIS in advance of systemic thrombolysis via rtPA and/or EVT [11–16].

Recent studies suggest that FDCT might support outcome prediction after EVT in AIS patients [17, 18]. However, those studies are limited to a small patient cohort and the definition of infarct demarcation was restricted to hyperattenuated tissue changes.

Thus, we aimed to assess FDCT-based outcome prediction in AIS patients after EVT on a large cohort of patients and including all radiological pattern of infarct demarcation using data from the Stroke Research Consortium in Northern Bavaria (STAMINA)—a longitudinal cohort study which contains patients with ischemic stroke admitted to the University Hospital Erlangen (Germany).

## Materials and methods

### Patients

All patients were recruited from the STAMINA (Stroke Research Consortium in Northern Bavaria; www.clinicaltrials.gov; NCT04357899) database, a longitudinal cohort study which contains patients with ischemic stroke admitted to the University Hospital Erlangen, Germany, between January 01, 2006, and June 30, 2019. The study was approved by the local institutional review board, Friedrich-Alexander-University Erlangen-Nuremberg, Germany (Registration No. 62_21B). Patients or legal representatives provided informed consent unless waived by the review board.

This subanalysis contains data from 517 patients who fulfilled the following inclusion criteria:

1. Admission between January 01, 2015, and June 30, 2019 (due to internal chances in diagnostic and therapeutic processes)
2. Complete and continuous clinical and radiological work-up, including initial non-invasive diagnostic, follow-up CT after 24 h, the National Institutes of Stroke Scale (NIHSS) at admission and pre-stroke modified Rankin Scale (mRS) as well as mRS at 90 days
3. AIS and LVO of the anterior circulation, namely the proximal and distal internal carotid artery (ICA) as well as the M1 and M2 segments of the middle cerebral artery (MCA)
4. Successful recanalization (mTICI ≥ 2B)
5. FDCT conducted after EVT with reliable diagnostic quality judged by two experienced neuroradiologists (MK and PH)

**Fig. 1:**
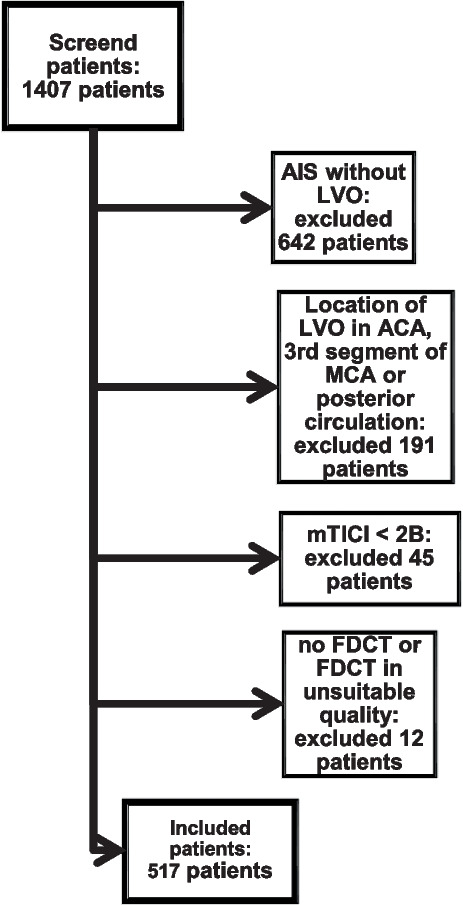
Flow chart of inclusion criteria of this study

A flow chart of the inclusion criteria of this study is presented in Fig. 1. There was no preselection regarding the type of thrombectomy, the content of the FDCT or the clinical course of the individual patient after EVT.

### FDCT

Immediately after EVT, FDCT was conducted for all 517 patients. This examination was performed on dedicated angiographic systems (Axiom Artis dBA or Axiom Artis Zee Zeego; Siemens Healthineers, Forchheim, Germany) using the 20sDR-H programme (DynaCT, Siemens AG Healthcare Sector, Forchheim, Germany; acquisition time 20 s, matrix 512, projection on 30 × 40-cm flat-panel size, total angle 217°). Postprocessing was done in an optimised soft tissue kernel (W380 C80). Secondary reconstructions were performed in transversal angulation (angulated after the base of the skull) with a standardised slice thickness of 4.8 mm and no gap.

### Image analysis

Radiological analysis was conducted using syngo.via software (syngo.via, Siemens Healthineers, Erlangen, Germany) under optimised diagnostic conditions and with consent by 2 experienced neuroradiologists (PH and MK) with 8 and 9 years of neurointerventional experience. Images were evaluated for presence of infarct demarcation. Demarcation was grouped in appearance on imaging (hypoattenuated vs. hyperattenuated vs. mixed), in location of the demarcation (basal ganglia vs. white matter vs. cortical) and according to ASPECTS. A volumetric analysis of the demarcation was also manually performed by defining the demarcation slice by slice and adjusting this result with the slice thickness. The Alberta Stroke Programme Early CT Score (ASPECTS) was applied to all datasets manually including all affected areas regardless of attenuation pattern [10]**.**

### Data analysis

For further analysis regarding a potential association between FDCT and clinical outcome, patients were grouped according to clinical outcome using a mRS shift analysis. Favourable clinical outcome was defined as shift in mRS (Δ mRS) from pre-admission to follow-up after 90 days of ≤ 1, whereas an unfavourable course was defined as a shift of ≥ 2.

Statistical analyses were done using commercially available software (IBM® SPSS® Statistics Version 28, Chicago, IL, USA). Normal distribution of continuous variables was performed using the Kolmogorov-Smirnov test. Normal distributed parameters are presented as mean ± standard deviation. In the absence of a normal distribution, we performed the Mann-Whitney *U* test; these data are demonstrated as median and interquartile range.

For the analysis of normal distributed parameters, we used the Wilcoxon test. For dichotomized variables, the chi-square test was used to compare between outcome groups. Spearman’s rank correlation coefficient was applied for further analysis of this correlations.

Statistical significance was considered for a *p* value of less than 0.05.

Multivariable regression analysis was applied to determine factors associated independently with the outcome. This analyse involved all clinical and radiological data available timely until the FDCT was conducted such as patient data (e.g. age, gender, mRS prior to admission), stroke-related clinical information (e.g. NHISS, time window), radiological data (e.g. ASPECTS, side and location of LVO) and therapeutic data (e.g. i.v. thrombolysis, EVT) as well as the above-mentioned results from the FDCT. A full overview is given in Table 1.

**Table 1:** Demographic and clinical characteristics

	Univariable analysis				Multivariable analysis	
Demographic and clinical characteristics	All patients (*n* = 517)	Patients with favourable outcome (Δ mRS) (*n* = 128)	Patients with unfavourable outcome (Δ mRS) (*n* = 389)	*p* value	OR (95% CI)	*p* value
Age (years)^†^	71.52 ± 13.73	69.16 ± 14.76	72.29 ± 13.31	0.052	0.001 (− 0.003–0.005)	0.641
Gender, female*	289 (55.9%)	73 (57%)	216 (55.5%)	0.766	0.012 (− 0.093–0.117)	0.821
LVO on the left side*	262 (50.7%)	68 (53.1%)	194 (49.9%)	0.54	0.029 (− 0.07–0.129	0.562
LVO of the ACI/MCA*	164 (31.7%)/353 (68.3%)	27 (21.1%)/101 (78.9%)	137 (35.2%)/252 (64.8%)	0.008	− 0.033 (− 0.083–0.017)	0.2
mRS prior to admission^‡^	0 (0–2)	1 (0–3)	0 (0–1)	0.001	Not included	
NHISS at admission^‡^	15 (11–19)	14 (9–18)	16 (12–20)	0.001	0.01 (0.003–0.018)	0.008
Onset-to-admission time (in minutes)^‡^	140 (67–372)	94.5 (55.8–311.3)	149 (70–411)	0.031	− 9.8 × 10^-7^ (0.0–0.0)	0.99
Initial ASPECTS^‡^	9 (7–10)	9 (8–10)	9 (7–10)	0.034	− 0.003 (− 0.032–0.026)	0.821
i.v. thrombolysis*	350 (67.7%)	89 (69.5%)	261 (67.1%)	0.635	− 0.027 (− 0.133–0.079)	0.619
Mechanical thrombectomy*	470 (90.9%)	111 (86.7%)	359 (92.3%)	0.008	0.34 (− 0.002–0.682)	0.052
Infarct demarcation in DynaCT in ml^‡^	2.25 (0–13.4)	0 (0–43)	4 (0–19)	0.001	− 0.002 (− 0.004–0.0)	0.126
FDCT-ASPECTS	9 (7–10)	9 (9–10)	9 (7–10)	0.001	− 0.055 (− 0.102–0.008)	0.022
Infarct demarcation after 24 h^‡^	27.1 (7–95.5)	9 (1–33)	40 (12–120)	0.001	Not included	
ICH after 24 h*	47 (9.1%)	6 (4.7%)	41 (10.5%)	0.128	Not included	
mRS after 90 days^‡^	4 (2–5)	2 (1–3)	4 (3–6)	0.001	Not included	

*LVO* large vessel occlusion, *mRS* modified Rankin Scale, *NHISS* National Institutes of Health Stroke Scale, *ASPECTS* Alberta Stroke Program Early Computed Tomography Score, *i.v.* intravenous, *ml* millilitre, *h* hours

^†^Mean ± standard deviation

^*^n (%)

^‡^Median (interquartile range 25th–75th percentile)

For items with a *p* value of < 0.05, a receiver operating characteristic (ROC) curve analysis with calculation of the area under the curve (AUC) was performed to determine the predictive power. To determine an optimal cut-off point in FDCT-ASPECTS for a favourable outcome, the Youden index was applied.

## Results

### Baseline characteristics

A total of 517 patients were included in the final analysis (289 women and 228 men, 71.52 ± 13.73 years, NHISS 15 (11–19)). AIS location was ICA (proximal and distal; 31.7%) and MCA (M1 and M2; 68.3%). A total of 262 (50.7%) patients had a left-sided AIS (Table 1).

**Fig. 2:**
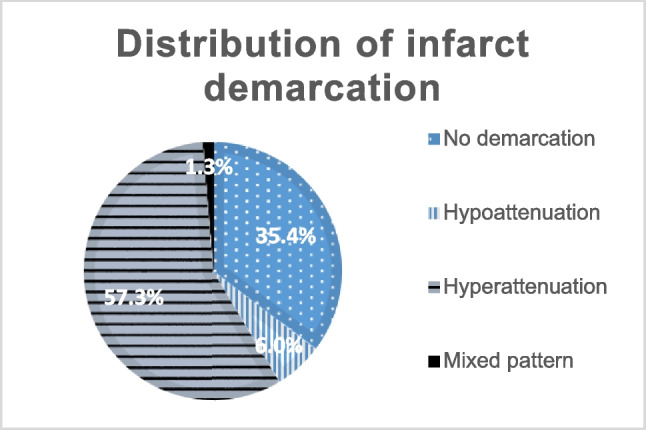
Distribution of infarct demarcation pattern

The FDCT analysis showed an absence of demarcation in 183 cases (35.4%). Hypoattenuation was visible in 31 (6%) whereas hyperattenuation was seen in 296 images (57.3%). A mixed pattern of demarcation was seen in 7 cases (1.3%) (Fig. 2, Picture 1).﻿

**Picture 1:**
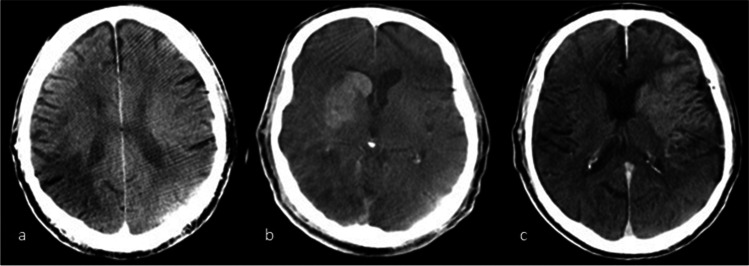
Examples of FDCT with different patterns of demarcation. (**a**) Purely hypoattenuated pattern (ASPECTS 9). (**b**) Purely hyperattenuated pattern (ASPECTS 7)*.* (**c**) Mixed pattern (ASPECTS 2): hyperattenuation in Ncl. caudatus, putamen, internal capsule, insular cortex, M1/2, and M5 (representative slice not shown) hypoattenuation in M3 (nomenclature after Barber et. al. [11])

The postinterventional FDCT-ASPECTS and the initial ASPECTS based on non-enhanced CT were 9 (7–10) respectively with a Spearman rank correlation coefficient of 0.304 (*p* < 0.001). (Fig. 2)

### Patient outcome

**Fig. 3:**
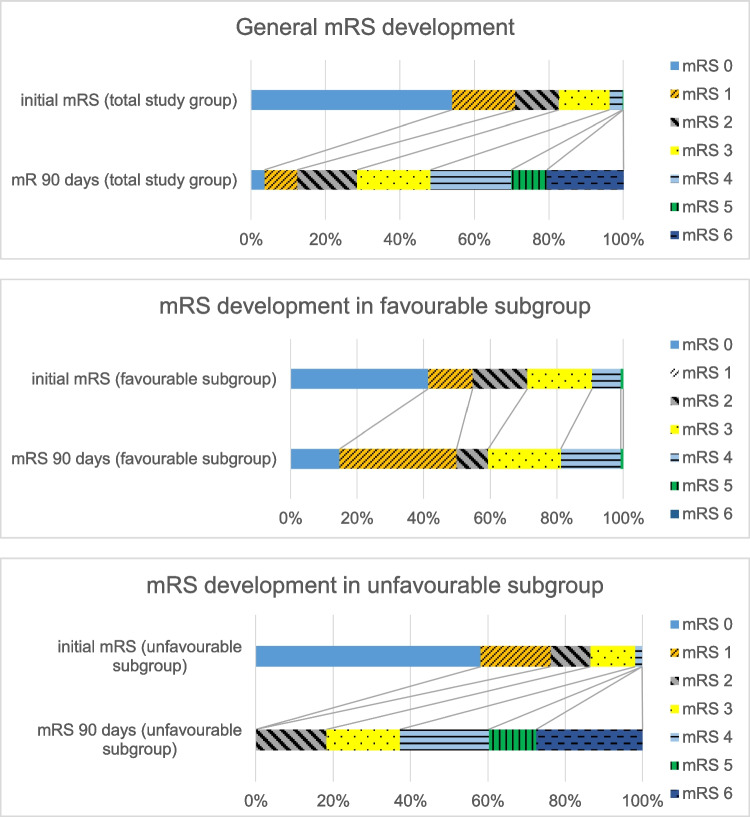
Development of mRS from pre-hospitalisation to 90 days after discharge for the total study group (**a**), the subgroup with favourable development (**b**) and the subgroup with unfavourable development (**c**)

One hundred twenty-eight (24.8%) cases were classified as having a favourable outcome (Δ mRS) from pre-admission to 90 days after discharge (Table 1, Fig. 3).

**Fig. 4:**
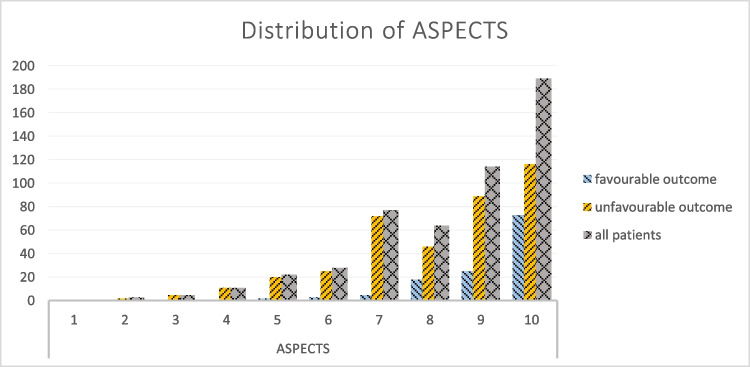
Distribution of FDCT-ASPECTS for the total study group, the subgroup with favourable development and the subgroup with unfavourable development

The subgroup with favourable mRS shift showed a statistically significant difference in FDCT-ASPECTS compared to the subgroup with unfavourable outcome (median ASPECTS 10 (10–9) vs. median ASPECTS 9 (10–7); *p* = 0,001). Mulitvariable regression analysis revealed FDCT-ASPECTS (OR 0.234, 95% CI − 0.102–0.008, *p* = 0.022) along with the NHISS at admission (OR 0.169, 95% CI 0.003–0,018, *p* = 0,008) as independent factors for a favourable development. Further details are illustrated in Table 1 and distribution of FDCT-ASPECTS is shown in Fig. 4.

**Fig. 5:**
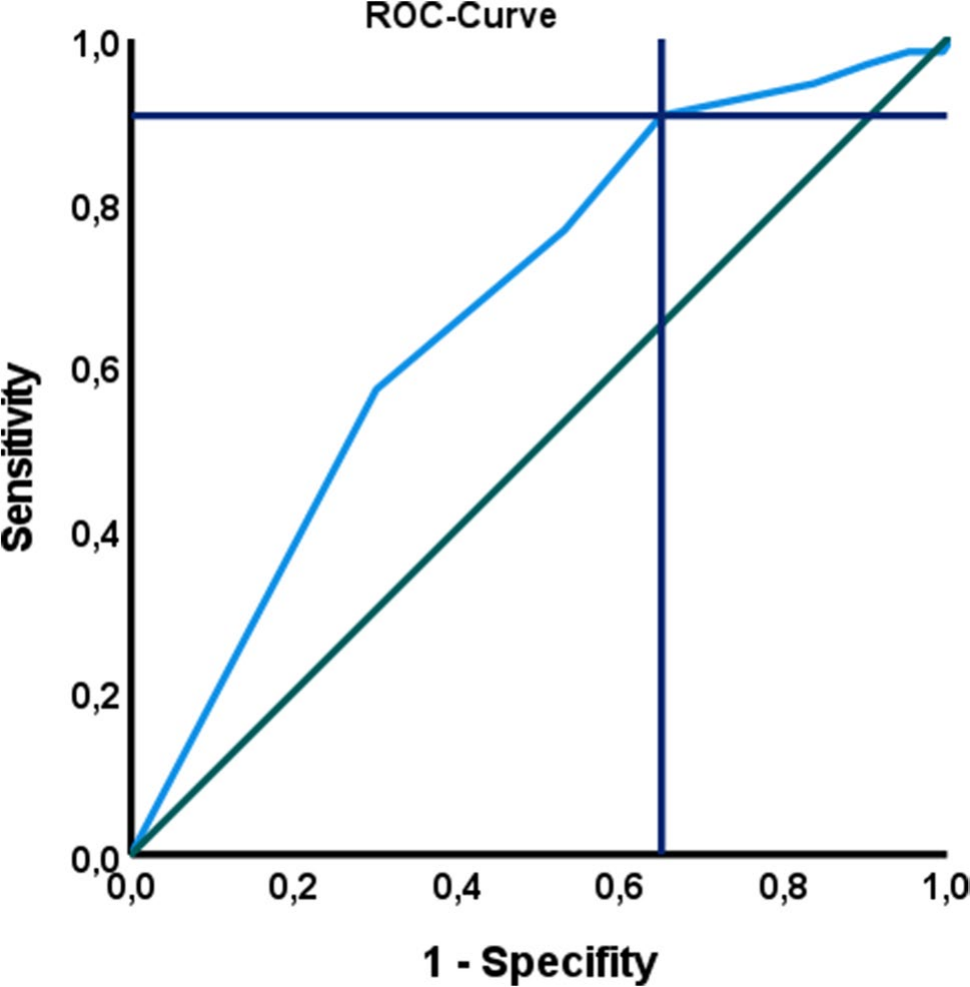
ROC curve analysis for FDCT-ASPECTS

To define a cut-off value for favourable mRS shift, we performed a ROC curve analysis. The AUC of FDCT-ASPECTS was 0.671 (95% CI 0.619–0.722, *p* < 0,001). A cut-off value for favourable mRS shift was identified at an ASPECTS ≥ 8 (sensitivity 90.6%, specificity 35%, Fig. 5).

## Discussion

The results of our study demonstrate a statistically significant association between FDCT-ASPECTS and the clinical outcome in patients with AIS due to LVO of the anterior circulation successfully treated with EVT.

As a source of parenchymal imaging, non-contrast whole-brain flat-detector CT (FDCT) is already established in the workflow of EVT in many institutes [4, 5, 8].

On the other hand and based on conventional non-enhanced CT scans, ASPECTS as a scoring system is already an inherent part of initial stroke diagnostics prior to EVT [11–14, 16]. Therefore, in transferring ASPECTS on FDCT, we applied a well-established and easy-to-use scoring system onto a different, but also well-established imaging modality.

Our study transfers FDCT-ASPECTS onto a large group of patients successfully treated with EVT. In addition to previous studies on this subject, we extended the definition of infarct demarcation to all possible patterns and adjusted the clinical outcome using mRS-shift from pre-admission to 90 days [19].

Previously, Chung et al. analysed the pattern of infarct demarcation in FDCT with respect to the clinical course. This study limited its results to a hyperattenuated form of demarcation and concentrated its focus on analysing the regional pattern of demarcation (striatal vs. cortical vs. combined). In applying ASPECTS on FDCT, we sharpened the regional aspect of demarcation compared to Chung et al. [18].

Furthermore, Baek et al. also described a direct connection between a good FDCT-ASPECT and a favourable clinical outcome in the long term. Our results and the findings of Baek et al. are consistent in demonstrating the prognostic value of the FDCT-ASPECTS for the clinical outcome as well as in postulating the same cut-off point for FDCT-ASPECTS for a more favourable clinical curse [17].

Baek et al. as well as Chung et al. did focus on hyperattenuated areas for resurge. Schneider et al. described a close relationship between these hyperattenuated lesions and infarcted tissue [20].

Contrary to Baek et al. and Chung et al., our study has two major differences in design.

First, we extended the included pattern of infarct demarcation. The second difference lies in a slightly differing and—in our view—more accurate definition of favourable vs. unfavourable outcome.

The first difference lies in a less restricted definition of infarct demarcation. In FDCT in general, hypoattenuation as a pattern of infarct demarcation is also detectable and seen on a regular base in clinical practise [7]. To reflect reality, we therefore integrated hypoattenuated and hyperattenuated as well as mixed pattern of demarcation in our study and treated those patterns equally for assessment of ASPECTS.

The second difference lies in including patients with more severe pre-stroke disability in our study. In clinical practise and according to national guidelines, patients with pre-existing disability and beyond a pre-mRS of 3 are also considered for and treated with EVT [21]. Therefore, we adjusted the mRS at 90 days with the pre-hospitalisation mRS and demanded a stable or only 1 point increasing mRS as favourable outcome.

Our results are consistent with Baek et al. regarding the categorisation of FDCT-ASPECTS as an independent prognostic factor for the outcome as well as in the actual placement the cut-off point [17].

Compared to Baek et al und Chung et al., the impact of our results is even higher as we have not only the largest study cohort (517 patients) but extended also the included pattern of infarct demarcation to all possible forms [17, 18].

Previously, it was difficult to detect early infarct signs in FDCT due to its lower contrast resolution in comparison to conventional CT images [22]. However, due to improvements in flat-detector technic and software, it becomes more common to detect not only hyperattenuation resulting from contrast staining but also a mixed or poorly hypoattenuated pattern of infarct demarcation [7, 23].

As the distribution in the pattern of demarcation in our study population demonstrates, a poorly hypoattenuated infarct demarcation in the directly postinterventrional setting is still numerical minor to other forms of demarcation but may still be encountered. Therefore, we decided to incorporate any form of possible infarct demarcation in our analysis. The dominance of the hyperattenuated pattern in FDCT after EVT can be blamed only partially on technical characteristics of FDCT and must be also seen in the context of the previously conducted EVT and application of contrast medium.

## Limitations

Our study has several limitations: first, our study design is retrospective and single centered, which bears the risk of a selection bias.

Second, we excluded patients with an unsuccessful EVT (meaning TICI < 2B) as this represents a criterion for unfavourable outcome in its own. A progression of demarcation after ending any attempts of restoring cerebral perfusion is very likely, and consequently, FDCT would have not been representative [24].

Third, we assessed the ASPECTS manually. It has been shown that software-based ASPECTS evaluation is much more replicable and reliable [25]. However, currently, an automized approach of parenchymal evaluation on non-enhanced FDCT-data is not commercially available. Due to technical differences between conventional CT and FDCT, today’s commercial available software is not valid for use on FDCT images [26, 27]. We compensated this in assessing FDCT and applying ASPECTS as a team of experienced neuroradiologists similar to Maier et al. [7].

## Conclusion

In consent with previous studies, we found FDCT-ASPECTS a highly reliable prognostic tool regarding the outcome after LVO and EVT. Thus, FDCT after EVT might extend current concepts of prognostic evaluation regarding the long-term outcome of AIS patients.

## Declarations

### Data availability

The datasets generated and analysed during the current study are available from the corresponding author on reasonable request.
